# Diversity of the *Epsilonproteobacteria* Dsb (disulfide bond) systems

**DOI:** 10.3389/fmicb.2015.00570

**Published:** 2015-06-09

**Authors:** Katarzyna M. Bocian-Ostrzycka, Magdalena J. Grzeszczuk, Lukasz Dziewit, Elżbieta K. Jagusztyn-Krynicka

**Affiliations:** Department of Bacterial Genetics, Faculty of Biology, Institute of Microbiology, University of WarsawWarsaw, Poland

**Keywords:** Dsb proteins, disulfide bonds, cytochrome c biogenesis, *Epsilonproteobacteria*, *Campylobacter*, *Helicobacter*, *Arcobacter*, *Wolinella*

## Abstract

The bacterial proteins of the Dsb family—important components of the post-translational protein modification system—catalyze the formation of disulfide bridges, a process that is crucial for protein structure stabilization and activity. Dsb systems play an essential role in the assembly of many virulence factors. Recent rapid advances in global analysis of bacteria have thrown light on the enormous diversity among bacterial Dsb systems. While the *Escherichia coli* disulfide bond-forming system is quite well understood, the mechanisms of action of Dsb systems in other bacteria, including members of class *Epsilonproteobacteria* that contain pathogenic and non-pathogenic bacteria colonizing extremely diverse ecological niches, are poorly characterized. Here we present a review of current knowledge on *Epsilonproteobacteria* Dsb systems. We have focused on the Dsb systems of *Campylobacter* spp. and *Helicobacter* spp. because our knowledge about Dsb proteins of *Wolinella* and *Arcobacter* spp. is still scarce and comes mainly from bioinformatic studies. *Helicobacter pylori* is a common human pathogen that colonizes the gastric epithelium of humans with severe consequences. *Campylobacter* spp. is a leading cause of zoonotic enteric bacterial infections in most developed and developing nations. We focus on various aspects of the diversity of the Dsb systems and their influence on pathogenicity, particularly because Dsb proteins are considered as potential targets for a new class of anti-virulence drugs to treat human infections by *Campylobacter* or *Helicobacter* spp.

## Introduction

**Figure 1:**
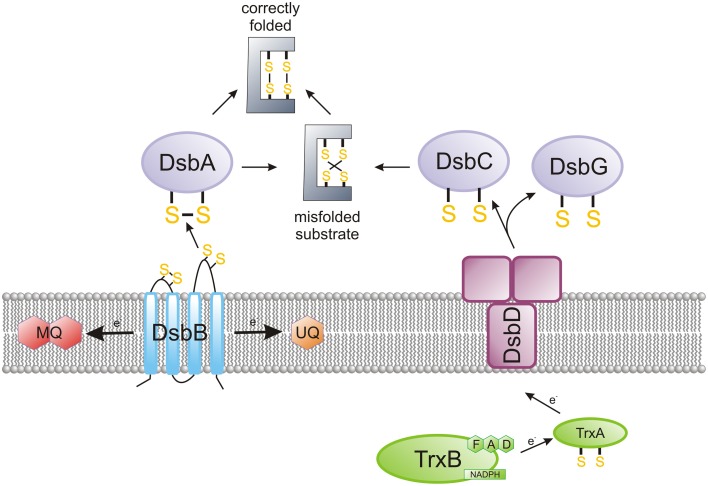
**The main features of the oxidized protein folding in** ***Escherichia coli***. The bacterial proteins of the Dsb family catalyze the formation of disulfide bridges, a post-translational modification in many extracytoplasmic proteins. EcDsbA is a TRX family member, which forms disulfides in a consecutive manner on polypeptide chains that are entering the periplasm. EcDsbA is reoxidized by integral membrane protein EcDsbB, which catalyzes the electron transfer to the respiratory chain. EcDsbC ensures the rearrangement of improperly introduced disulfides. In *E. coli*, DsbC is kept in the reduced form by an integral membrane protein, DsbD, which catalyzes the transfer of electrons from the cytoplasm to the periplasm. The *Campylobacter* Dsb system is more complex than the *E. coli* system in terms of the number of elements included. Generally, *Campylobacter* species contain two DsbA and two DsbB proteins. However, several differences are observed not only among various species of the *Campylobacter* genus but also between strains of the same species (see **Figures 2, 4**). *Helicobacter pylori*, the best characterized member of the *Helicobacter* genus, does not encode the classical DsbA/DsbB oxidoreductases. Instead, it possesses an unusual pair of proteins involved in disulfide bond formation—periplasmic HP0231 (DsbK), with a dimeric structure that resembles EcDsbG/C, and its membrane partner HpDsbI (HP0595), a protein with a β-propeller structure. The mechanism for the rearrangement of incorrectly introduced disulfides was not examined in *Campylobacter* and *Helicobacter* cells.

The oxidation reaction between two cysteine thiol groups, combined with the release of two electrons, results in the formation of a disulfide bond. This bond formation is a rate-limiting step in the protein folding process, and it is catalyzed by proteins of the Dsb (*d*i*s*ulfide *b*ond) system. This post-translational protein modification takes place in oxidative environments: either in the periplasm for Gram-negative bacteria or the space between the cytoplasmic membrane and the cell wall for Gram-positive bacteria (Hatahet and Ruddock, 2013). The formation of disulfide bonds (Dsb) plays a key role in bacterial virulence, which often depends on cysteine-rich, extracytoplasmic proteins (Heras et al., 2009, 2015; Denoncin and Collet, 2013). A combination of microbiological, biochemical, biophysical and proteomic approaches has yielded a detailed characterization of the Dsb protein network for the model microorganism, *Escherichia coli* (EcDsb proteins). In general, as shown on Figure 1, there are two, mostly antagonistic, metabolic pathways acting in the *E. coli* periplasm: an oxidation pathway and an isomerization/reduction pathway (Messens and Collet, 2006; Gleiter and Bardwell, 2008; Ito and Inaba, 2008; Depuydt et al., 2011).

The first reaction (catalyzed by EcDsbA and EcDsbB) is responsible for the formation of disulfide bonds in the newly synthesized proteins, just after they cross the cytoplasmic membrane (Kadokura and Beckwith, 2009). As this process occurs in a non-selective way, a second reaction (driven by EcDsbC and EcDsbD) rearranges improperly introduced disulfide bonds (Gruber et al., 2006). A large-scale bioinformatic analysis of bacterial genomes to search for Dsbs homologs, in conjunction with detailed functional and structural studies of DsbAs, has revealed that the pathways for disulfide bond formation within the bacterial kingdom are extremely diverse. The sets of Dsb proteins involved in the oxidative pathway varies, depending on the microorganism. The differences are observed not only between various bacterial genera and species, but also between bacterial strains of the same species. For instance, certain bacterial genomes encode multiple DsbAs with different substrate specificities, or multiple DsbBs; some other bacterial genomes possess only DsbA homologs; and others encode neither DsbA nor DsbB (Heras et al., 2009). In some microorganisms, the bacterial homolog of the vitamin K epoxide reductase (VKOR) plays a DsbB role (Li et al., 2010; Landeta et al., 2015). There are also some bacteria that possess Dsb proteins that fold into a V-shaped, homodimeric molecule similar to EcDsbC and EcDsbG, but are involved in disulfide bond formation like monomeric DsbAs (Daniels et al., 2010; Roszczenko et al., 2012; Kpadeh et al., 2013). Additionally, there are some microorganisms that do not have any components of the Dsb system (Dutton et al., 2008).

The three-dimensional structures of many DsbA-homologous proteins have recently been determined. There are about 25 DsbA structures deposited in the Protein Data Bank (PDB) (April 2015). These crystallographic studies revealed that even though all DsbAs possess a common thioredoxin fold, containing the active site with a CXXC motif, they display numerous structural differences that influence their biochemical properties, including redox potential and substrate specificity (McMahon et al., 2014). The redox potential of the Dsb proteins is mainly dependent on both the XX dipeptide within the active site CXXC motif and a residue located upstream of the *cis*-proline loop (which is distant in the linear sequence but close to the CXXC in the three-dimensional structure) (Charbonnier et al., 1999; Lafaye et al., 2009; Ren et al., 2009). Most of the EcDsbA homologs (more than 70%) possess a CXXC motif identical to EcDsbA (CPHC). However, various combinations of amino-acids in the XX dipeptide of the CXXC motif have been observed among members of the DsbA family (Chivers et al., 1996; Quan et al., 2007; Heras et al., 2009). The diverse redox properties of the DsbAs, as well as other TRX-fold proteins, are assumed to be also determined by indirect interactions of polar residues with the side chain of the N-terminal catalytic cysteine residue (Rinaldi et al., 2009). Members of DsbA-family fall into two main DsbA classes, based on comparison of their structural and biochemical features (McMahon et al., 2014).

The epsilon subdivision of the Gram-negative *Proteobacteria* has two major orders: *Campylobacterales* and *Nautiliales*. This taxon has multiple genera and includes both pathogenic and non-pathogenic microorganisms. The pathogenic group mainly colonizes the gastrointestinal tracts of many animal species (including humans); the non-pathogenic group are mainly microorganisms that live freely in water. Some of these occupy exceptional ecological niches, such as deep-sea hydrothermal vents (Miroshnichenko and Bonch-Osmolovskaya, 2006; Nakagawa et al., 2007; Giovannelli et al., 2011). The most prominent representatives of human pathogens are members of the *Campylobacterales* order belonging to the genus *Campylobacter* and *Helicobacter*, and *Arcobacter* and *Wolinella* strains are now also being analyzed.

Human infection by *Campylobacter* constitutes an important public-health problem worldwide. The greatest threat comes from two species, *Campylobacter jejuni* and *Campylobacter coli*. Both are inhabitants of the chicken intestinal tract, and each is a major etiological agent of human gastroenteritis (Kirkpatrick and Tribble, 2011; EFSA, 2014). In some immunocompromised humans, infection by these species can result in severe autoimmune disorders such as Guillain-Barré or Miller-Fisher syndromes (Zilbauer et al., 2008). New members of *Campylobacter* species, such as *C. curvus* or *C. concisus*, have recently been identified as emerging human and animal pathogens (Silva et al., 2011).

The genus *Arcobacter*, closely related phylogentically to *Campylobacter*, contains pathogenic and non-pathogenic species isolated from various ecological niches. These microorganisms are now recognized as emerging zoonotic enteropathogens, and contaminated food or contaminated water are the major sources of human infections (Collado and Figueras, 2011).

Although numerous identified species of the *Helicobacter* genus colonize various vertebrates, most of global research has focused on two species: *Helicobacter pylori* and *Helicobacter hepaticus*, as they are important predisposing factors in gastric cancers in humans (Yamaoka and Graham, 2014; Segura-Lopez et al., 2015). Infection with *H. pylori* affects about half of the world's population, yet, its prevalence varies geographically. Currently, infections are highly prevalent in developing countries, but are disappearing in well developed countries. *H. pylori* infections induce both acute and chronic gastritis and peptic ulcers. *H. pylori* is also considered to be a high risk factor for the development of mucosa-associated lymphoid tissue lymphoma and adenocarcinoma of the stomach (De Falco et al., 2015). Based on results of clinical studies, the World Health Organization (WHO) has designated *H. pylori* infections as class I carcinogens (WHO, 1994). However, it should be pointed out that an *H. pylori* infection has two faces (Bocian and Jagusztyn-Krynicka, 2012). This bacterium has accompanied humans for at least 60,000 years (Linz et al., 2007). The prevalence of *H. pylori* infection has been decreasing over the past 50 years. At the same time an increased incidence of gastroesophageal reflux disease and esophageal adenocarcinoma, has been noted. Some studies also suggest an inverse correlation of *H. pylori* infection with childhood asthma or obesity. Thus, it is debatable whether we should consider this bacterium as a colonizer or as a pathogen (Cover and Blaser, 2009; Cid et al., 2013; Otero et al., 2014).

While *Wolinella succinogenes* is classified as a member of the *Helicobacteriaceae*, phylogenetic studies show that it is an “intermediate” between the *Campylobacteriaceae* and *Helicobacteriaceae* families (Baar et al., 2003).

**Figure 2:**
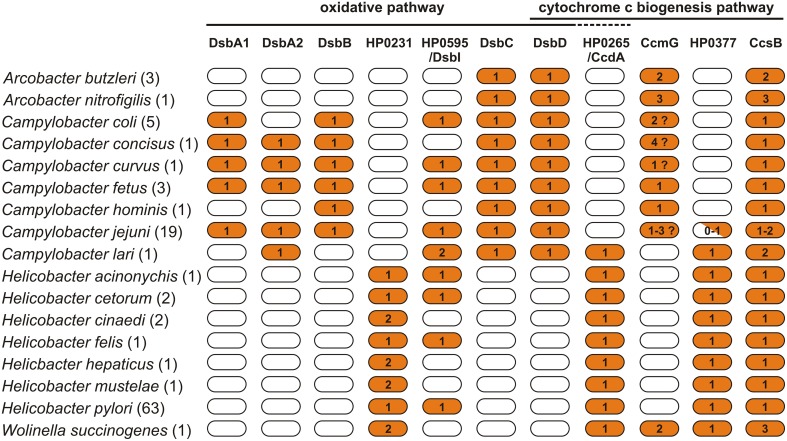
**Distribution of proteins of the Dsb family in** ***Arcobacter*** **spp.,** ***Campylobacter*** **spp.,** ***Helicobacter*** **spp., and** ***Wolinella succinogenes*** **DSM 1740 proteomes**. For the analysis, 107 bacterial proteomes were screened for the presence of 11 proteins involved in the oxidative pathway and cytochrome c biogenesis pathway. The exact number of proteomes used for each species is presented in parenthesis next to specie's name. The BLASTp search vs. a manual curated database, including 34 reference proteins, was applied. For the analysis, the following cutoff values were applied (*e*-value < 10^−10^ and amino acid sequence identity of at least 25%). Numbers indicate the copy number of proteins in the particular proteomes.

In this review we present progress that has recently been made to unravel the intricate details of how the Dsb systems of pathogenic *Epsilonproteobacteria* function. We focus on the roles the Dsb systems play in oxidative protein folding and cytochrome c biogenesis, and we also present potential benefits in therapy that may arise from accumulated knowledge about Dsb systems. The review also encompasses data on the *in silico* analysis of Dsb proteins of 107 representatives *Epsilonproteobacteria*, including: (i) *Arcobacter* spp. (4 strains), (ii) *Campylobacter* spp. (31 strains), (iii) *Helicobacter* spp. (71 strains), and *W. succinogenes*. The results of our *in silico* analysis (Figure 2, Table S1) summarize the current knowledge and add some novel observations concerning diversity of the *Epsilonproteobacteria* Dsb systems.

## Brief characteristics of the *E. coli* oxidative and isomerization Dsb system

In the *E. coli* proteome, the periplasmic DsbA and its partner, an inner-membrane protein DsbB, are encoded by two monocistronic units located in distinct chromosomal *loci*. DsbA is a 21-kDa monomeric protein in the TRX family, and it directly donates its disulfide bond that is present in the active site to reduce protein substrates. This reaction is conditioned by the presence of two highly conserved motifs: the CXXC (CPHC) active site within the TRX fold and the *cis*-proline loop. Both motifs are responsible for numerous physico-chemical properties of the DsbA enzyme, and they play a significant role in its folding and stability, as well as in DsbA's interaction with its substrate(s) and its redox partner, DsbB (Kadokura et al., 2004, 2005). In *E. coli*, DsbA is converted back to the oxidized form by the inner membrane protein DsbB, which has four transmembrane segments and two periplasmic loops, each containing one pair of conserved, essential catalytic cysteine residues. DsbB donates electrons to either ubiquinone during aerobic growth or to menaquinone during anaerobic growth. In contrast to the non-specific oxidoreductase DsbA, DsbB appears to be a very specific enzyme. Under physiological conditions, it is able to oxidize only the reduced form of DsbA, by rapid disulfide exchange (Tapley et al., 2007; Pan et al., 2008).

In *E. coli*, the periplasmic protein disulfide isomerase, DsbC is involved in rearrangements of incorrectly paired cysteines, so it is required *in vivo* for full activity of a handful of proteins that contain at least one non-consecutive disulfide bond (Hiniker and Bardwell, 2004). Its X-ray structure has been solved and shows that EcDsbC is a dimeric protein with two 23.3 kDa monomers arranged into a V-shaped homodimeric molecule (McCarthy et al., 2000). Each monomer forms an arm of the V and consists of two domains: a C-terminal catalytic domain with a TRX fold, and an N-terminal dimerization domain. The dimerization of DsbC is crucial for its activity, as it leads to the formation of the substrate binding domain (Segatori et al., 2004; Arredondo et al., 2009). The C- and N-terminal domains are connected via a long α-linker. There are four conserved cysteine residues in each monomer. Two of these residues are arranged in a CXXC motif and are essential for the oxidoreductase activity of the protein (Hiniker et al., 2007).

The *E. coli* periplasmic oxidoreductase, DsbG, was for a long time considered to be a member of the isomerization/reduction pathway. Although the structure of DsbG resembles that of DsbC, significant structural differences between EcDsbC and EcDsbG were noticed (Heras et al., 2004). Additionally, EcDsbG it is not active in the insulin reduction assay and does not catalyze oxidative refolding of incorrect disulfides present in model protein substrates such as scrambled hirudine or scrambled RNase. Examined *in vivo*, its isomerizing activity is poor as compared with EcDsbC (Bessette et al., 1999; Hiniker et al., 2007). Depuydt et al. showed that EcDsbG interacts with periplasmic proteins containing a sole cysteine (Depuydt et al., 2009). Thus, it is generally accepted that DsbG is mainly involved in the control of the cysteine sulfenylation level, which protects single cysteine residues from oxidation to sulfenic acid.

In *E. coli*, DsbC and DsbG are kept in the reduced form by an integral membrane protein, DsbD, that catalyzes the transfer of electrons from the cytoplasm to the periplasm. The *E. coli* DsbD consists of eight transmembrane segments (β domain), an N-terminal domain (α domain) and a C-terminal domain (γ domain). The N- and C-terminal domains both face the periplasm. It has been established that the electrons flow from NADPH in the cytosol, via the TRX-1, β, γ, and α domains of DsbD, and then to various extracytoplasmic proteins (Stirnimann et al., 2006). Figure 1 presents key elements of the *E. coli* oxidative protein folding. Several excellent reviews about functioning of the *E. coli* Dsb system have recently been published (Shouldice et al., 2011; Berkmen, 2012; Denoncin and Collet, 2013; Hatahet et al., 2014).

## Oxidative and isomerization Dsb pathways of *Campylobacter* spp. and *Arcobacter* spp.

Phylogenetic comparisons based on 16sRNA have divided members of the *Campylobacter* genus into two major groups: the thermotolerant species (group I) and the non-thermotolerant species (group II) (Nothaft et al., 2012; Porcelli et al., 2013). Members of both groups were included in our analysis. Group I is represented by *C. jejuni*, *C. coli*, and *C. lari*; whereas group II is represented by *C. fetus*, *C. curvus*, *C. concisus*, and *C. hominis*.

We performed *in silico* analysis of the proteomes of 31 members of the *Campylobacter* genus, with respect to the presence of Dsb oxidoreductases (Figure 2). A BLASTp search, vs. a manually curated database including 34 reference proteins, was applied. Among the Dsb-like proteins identified, the oxidative folding activity was experimentally investigated for only two model *C. jejuni* strains: 81-176 and 81116 (Raczko et al., 2005; Grabowska et al., 2011, 2014). Both strains were isolated from human having campylobacteriosis and are commonly used for basic as well applied research by many laboratories (Palmer et al., 1983; Korlath et al., 1985). Genome sequences of both strains were determined (Hofreuter et al., 2006; Pearson et al., 2007). The *C. jejuni* 81116 genome is slightly smaller than that of C. *jejuni* 81-176 (1,628,115 bp and 1,641,481 bp, respectively). Sequencing of the *C. jejuni* 81-176 genome revealed some striking genetic attributes conditioning its high virulence (Hofreuter et al., 2006). It also contains two resident plasmids, pVir and pTet whose products affect virulence (Bacon et al., 2000, 2002; Batchelor et al., 2004). *C*. *jejuni* 81116 has small numbers of hypervariable G tracts so it is genetically stable and considered to be appropriate for the genetic research (Manning et al., 2001; Pearson et al., 2007).

The knowledge concerning the *Campylobacter* Dsb isomerization pathway is extremely limited and comes only from *in silico* analysis. The analyses performed in this work indicated that all *Campylobacter* strains contain homologs of EcDsbC and EcDsbD, two proteins that are potentially involved in the isomerization process (Figure 2, Table S1). The next part of this section presents data concerning the *Campylobacte*r Dsb oxidative pathway.

The Dsb oxidative pathway of *C. jejuni* (CjDsb) differs from the well-characterized oxidative pathway of *E. coli*. The *E. coli* pathway has two proteins, DsbA and DsbB, that are encoded by two monocistronic operons located far-apart on the chromosome. In C. *jejun*i 81116 and *C. jejuni* 81-176, two model organisms of C. *jejuni*, the Dsb oxidative pathway consists of four extracytoplasmic proteins. Two are soluble periplasmic proteins (CjDsbA1 and CjDsbA2), and the other two (CjDsbB and CjDsbI) are anchored in the inner membrane (Grabowska et al., 2011).

CjDsbA1 and CjDsbA2 share a high degree of sequence identity (47%); CjDsbA1 and CjDsbA2, respectively, have sequence identities of 24 and 28% with EcDsbA and 28.5 and 39% with EcDsbL (a homolog of EcDsbA present in some pathogenic *Enterobacteriaceae* (Grabowska et al., 2014). Both proteins have a strong, positively charged electrostatic patch above the active site, similar to that of EcDsbL but not EcDsbA (Grimshaw et al., 2008). However, CjDsbA1 and CjDsbA2 vary considerably with regard to their active sites, and in the charge distribution on the protein surface that is opposite from the active site. These differences are reflected in their phenotypic characteristics. CjDsbA1 is involved in cell motility and the autoagglutation process, and it is responsible for the formation of the disulfide bond of alkaline phosphatase, CjPhoX. The arylsulfotransferase CjAstA is the only substrate of CjDsbA2 identified so far, and it is not a substrate of CjDsbA1 (Grabowska et al., 2014). Similar relationships for multiple DsbAs in one bacterial proteome have also been described in other bacteria. The two redox protein pairs present in the proteome of *E. coli* UPEC CFT073 (EcDsbA/EcDsbB and the EcDsbL-EcDsbI) also differ in their substrate specificity and only the EcDsbL-EcDsbI redox pair plays a role in introducing disulfide bonds into AstA (Totsika et al., 2009). Another oxidative Dsb pathway with more than one DsbA is found in S. *enterica* sv. Typhimurium, which contains three DsbAs (i.e., SeDsbA, SeDsbL, and SeSrgA). Only the SeDsbL-SeDsbI redox pair restored full activity of AstA in a triple DsbA-homolog mutant (Heras et al., 2010). The hypothesis of a limited spectra of substrate for both of the *C. jejuni* DsbAs is also supported by the fact that neither CjDsbA1 nor CjDsb2 is active in insulin reduction (Grabowska et al., 2014).

The biochemical properties of the DsbA proteins are influenced by the cysteine-flanked dipeptide sequence of the CXXC motif and by the nature of the *cis*-Pro motif. The most common motif of bacterial DsbAs is CPHC paired with V*c*P. So, we compared the active site CXXC and *cis*-Pro motifs of the *Campylobacter* DsbAs. We found that all but one DsbAs of the group I members have the CIHC/CTHC motif paired with T*c*P, which is atypical for DsbA. The DsbA proteins of two species of group I that have only one DsbA also contain a CIHC motif in the active site paired with T*c*P. Group II members contain DsbA2 with CPFC, V*c*P motifs (close to that of the classical DsbA–CPHC, V*c*P), whereas the residues of the dipeptide in the CXXC motifs of their DsbA1 proteins vary considerably and are paired with either T*c*P or V*c*P (CIHC, T*c*P for *C. fetus*; CQHC, V*c*P for *C. curvus;* and CPHC, V*c*P for *C. concisus*) (Table S1).

**Figure 3:**
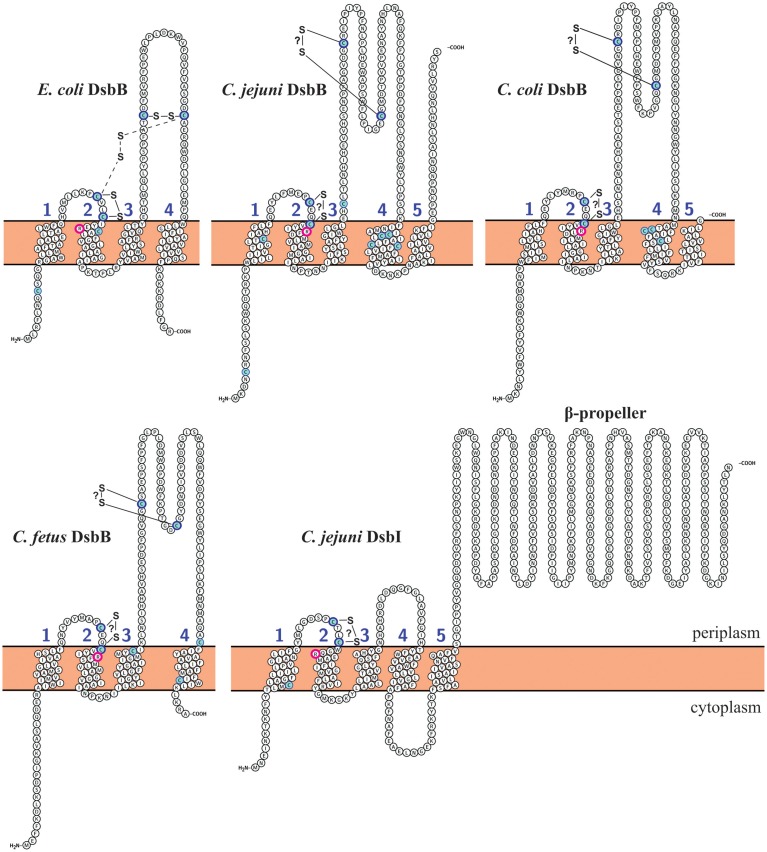
**DsbB and DsbI topological model predictions for** ***Campylobacter*** **species and comparison with EcDsbB (*****E. coli*****)**. *C. jejuni* DsbI model is identical as *C. lari* DsbI1, DsbI2, and *H. pylori* DsbI. Transmembrane topology predictions of DsbB and DsbI proteins were determined using Protter (http://wlab.ethz.ch/protter/#) (Omasits et al., 2014). Predictions were confirmed with TOPCONS (http://topcons.cbr.su.se/) (Bernsel et al., 2008, 2009; Viklund et al., 2008), PSIPRED (http://bioinf.cs.ucl.ac.uk/psipred/) (Buchan et al., 2013) and PredictProtein (http://predictprotein.org) (Rost et al., 2004). Disulfide bonding patterns were predicted using DiANNA software (http://clavius.bc.edu/~clotelab/cgi-bin/DiANNA/DiANNA.py) (Ferre and Clote, 2005). Cysteine residues are marked with blue. Conserved arginine residues are circled with pink. Intermediate disulfide bond between Cys41 and Cys140 in EcDsbB is mark with dashed lines. Disulfide bonds that are not confirmed experimentally are marked with question mark (?). All predictions were compared and manually corrected according to published data.

In *E. coli*, the activity of DsbA is restored by the action of EcDsbB, which provides its reoxidation. In EcDsbB, four cysteine residues, located in the periplasmic loops, play a crucial role in EcDsbA re-oxidation (Figure 3) (Kadokura and Beckwith, 2002). In *C. jejuni*, there are two homologs of EcDsbB: CjDsbB and CjDsbI. As was recently shown in *C. jejuni* 81-176 and 81116, only the CjDsbA2 activity is fully CjDsbB-dependent, and the possible cooperation between CjDsbA1 and CjDsbB is unclear. CjDsbI is not involved in either CjDsbA1 or CjDsbA2 activities (Grabowska et al., 2014). The “classical” EcDsbB consist of one domain, formed by four trans-membrane segments (TM1-TM4) (Inaba, 2008; Inaba and Ito, 2008). We have attempted to predict the toplogy of the DsbBs of representatives from group I and group II *Campylobacter*. We found, as previously published, that *C. jejuni* DsbB and also *C. coli* DsbB, both contain five membrane-spanning domains compared with four for EcDsbB; whereas the DsbBs of *C. fetus*, *C. concisus*, *C. curvus*, and *C. hominis* revealed the same topology as EcDsbB (four TMs). Regardless of the number of TMs present, all *Campylobacter* DsbBs contain the two pairs of conserved Cys residues, which are located within the periplasmic loops connecting TM segments 1–2 and 3–4, as is also seen in EcDsbB. DsbI—atypical DsbB-like protein is present in all *Campylobacter* proteomes, so far, analyzed. The role of DsbI in the oxidative Dsb pathway is still not completely understood. CjDsbI comprises two domains. The N-terminal domain consists of five transmembrane helices with a conserved oxidoreductase CXXC motif, and it exhibits significant sequence similarity to proteins from the DsbB family. It also contains a conserved Arg residue implicated in the process of transferring electrons (Kadokura et al., 2000). Both CXXC motif and Arg are located in the 1-2 periplasmic loop. A key difference between DsbI and “classical” DsbB proteins is the lack of the second pair of Cys residues. Instead, proteins of the DsbI family possess a C-terminal, periplasm-located domain with a beta-propeller structure, as shown in Figure 3 (Raczko et al., 2005). The role of the C-terminal domain of CjDsbI is still unknown. Proteins with a beta propeller fold, are characterized by extreme sequence diversity, despite similarities in their three-dimensional structures. In eukaryotic cells, they have different functions, whereas in prokaryotic cells they are often involved in redox reactions (Jawad and Paoli, 2002). *C. lari* does not contain DsbB, but instead it encodes two genes for DsbI, we also checked the topology of these two DsbI proteins. We found no differences between them by the method we used (Figures 2, 3, Table S1).

**Figure 4:**
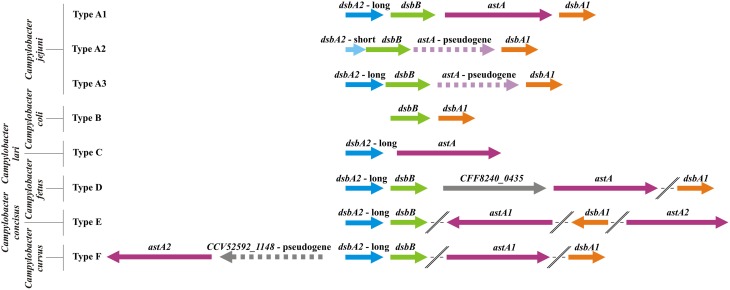
**Physical map presenting the genomic location of the** ***Campylobacter*** **Dsb oxidative pathway genes [*****dsbA1*** **(orange),** ***dsbA2*** **(blue),** ***dsbB*** **(green), and** ***astA*** **genes (purple)]**. The *astA* gene was also included as it is a part of the same transcriptional unit with *dsbA2* and *dsbB*, and its product is a substrate for DsbA2. Type A includes C. *jejuni* strains (A1: *C. jejuni* subsp. *jejuni* 81-176, 81116, ICDCCJ07001, MTVDSCj20, and *C. jejuni* 4031; A2: *C. jejuni* subsp. *jejuni* NCTC 11168 = ATCC 700819, 00-2425, 00-2426, 00-2538, 00-2544, IA3902, NCTC 11168-BN148, PT14, R14, and *C. jejuni* 32488; A3: *C. jejuni* subsp. *jejuni* S3 and M1, *C. jejuni* RM1221, *C. jejuni* subsp. *doylei* 269.97). Type B includes strains of *C. coli* species (*C. coli* RM5611, 15-537360, CVM N29710, RM1875 and RM4661). Type C is represented by one *C. lari* strain (*C. lari* RM2100). Type D includes three members of *C. fetus* species (*C. fetus* subsp. *fetus* 82-40, 04/554 and *C. fetus* subsp. *testudinum* 03-427). Type E is represented by one *C. concisus* strain (*C. concisus* 13826) and type F by one strain of *C. curvus* (*C. curvus* 525.92). The *dsbA1* and *astA* genes of *C. concisus, C. curvus*, and *C. fetus*, strains are located on the chromosome apart from *dsbA2dsbB* genes. Genes and intergenic regions are drawn to scale (based on underlined strains). Pseudogenes or fragmented genes are marked with dashed lines.

Our *in silico* analysis performed on all *C. jejuni* strains revealed that in respect to their genetic organization they can be classified into three types (Figure 4). The significant differences among the *Campylobacter* genomes with respect to *dsbA2* gene presence were observed. Type A1, represented by *C. jejuni* 81116 or 81-176, includes strains possessing two functional DsbA proteins (CjDsbA1 and CjDsbA2, respectively). In *C. jejuni* 81-176 and 81116 the *dsb* genes are organized into three operons. The monocistronic *dsbA1* operon is preceded by the *cjdsbA2-cjdsbB-cjastA* operon, in which *cjastA* codes for the Dsb substrate, arylsulfotransferase. The third operon, *cjdba-cjdsbI*, encodes for the DsbI and its accessory protein Dba, is distant from the other two on the chromosome (Grabowska et al., 2011; Dugar et al., 2013). Type A2 strains contain a truncated variant of *cjdsbA2* (encoding a putative TRX-like protein lacking the active CXXC motif), and they additionally lack a functional AstA. The strains classified as type A3 have already lost the functional *astA* gene, but they still encode a potentially active DsbA2. Regardless of the functionality of *dsbA2/astA*, the analyzed *dsb* genes are arranged into two operons, i.e., *cjdsbA2-cjdsbB-cjastA*, directly followed by a monocistronic unit *cjdsbA1* (Figure 4). Additionally two species that are also members of the thermotolerant group I, *C. coli* and *C. lari*, have lost one of the *dsbA2* genes (Figure 4—types B and C).

In contrast, in the genomes of *Campylobacter* spp. that belong to the non-thermotolerant group II (*C. fetus*, *C. concisus*, and *C. curvus*), only the *dsbA2* and *dsbB* genes constitute a putative operon. The *astA* gene, encoding a substrate of DsbA2, never forms an operon with *dsbA2/dsbB* and is located either in close proximity (*C. fetus* and *C. curvus*) to them or is present in a different chromosomal location (there are two copies of the *astA* gene in *C. concisus*). Additionally, the *dsbA1* genes of the group II *Campylobacter* spp., encoding oxidoreductases of potentially broad specificity, are located apart from the *dsbA2/dsbB* operon (Figure 4—types D, E, F). Our previous phylogenetical analysis showed that DsbA1-2, DsbB, and AstA all have very similar evolutionary histories, suggesting horizontal transfer of the entire DsbA1-2/DsbB2/AstA system from the *Campylobacter* genus to a common ancestor of the *Gammaproteobacteria* species, followed by its subsequent loss in most organisms of this clade. Moreover, phylogenetic analysis indicates that the divergence of CjDsbA1 and CjDsbA2 occurred after the horizontal transfer event (Grabowska et al., 2014).

All *Arcobacter* spp. genomes analyzed lack both a classical DsbA and DsbB members of Dsb oxidative pathway. However, they encode classical DsbC and DsbD, which are members of the Dsb isomerization pathway. The mechanism responsible for oxidative folding of proteins in this bacterium requires further investigation (Figure 2, Table S1).

## Introduction of the disulfide bond into extracytoplasmic proteins of *Helicobacter* spp. and *Wolinella succinogenes*

*H. pylori* Dsb oxidizing system is different from that operating in *E. coli* and in *Campylobacter* spp. that are phylogenetically related to *H. pylori*. The genome of *H. pylori* 26695 has 149 proteins containing the CXXC motif that is characteristic for the thiol:disulfide oxidoreductases identified to date. Only four of these proteins (HP0231, HP0377, HP0824, and HP1458) have a TRX fold (Kaakoush et al., 2007). Additionally, the *H. pylori* genome lacks classical DsbA and DsbB proteins, members of Dsb oxidative pathway, as well as classical DsbC and DsbD proteins, members of the Dsb isomerization pathway.

Our recent work (using biochemical and genetic approaches) led to the characterization of the first dimeric oxidoreductase (HP0231) that functions in an oxidizing pathway of *H. pylori* (Roszczenko et al., 2012). The knowledge about the role of HP0231 in cell physiology has been recently expanded by the data published by Lester et al. (2015). *H. pylori* isogenic *hp0231* mutant is non-motile and reveals some changes in the cell morphology. Additionally it is DTT-sensitive and shows reduced growth compared to the wild type parental strain (Roszczenko et al., 2012). Lack of HP0231 also affects *H. pylori* resistance to oxidative stress (Lester et al., 2015). HP0231 functioning in the oxidative protein folding was shown by proving its ability to complement the lack of DsbA in *E. coli* when delivered by a low-copy recombinant plasmid (Roszczenko et al., 2012) and by the observation that its over-expression in *E. coli* results in strong toxic effect (Lester et al., 2015). The structure of HP0231 has recently been solved (Yoon et al., 2011) and it appears to be a V-shaped protein like EcDsbC or EcDsbG. Two domains of HP0231, the catalytic and dimerization domains, resemble the corresponding domains of EcDsbG. However, several substantial structural differences between the two proteins have been shown. HP0231 and EcDsbG vary in the length of the linker helix and in the amino acid residues lining the V-shaped cleft (Yoon et al., 2011). Interestingly, the XX dipeptide from the active CXXC site of dimeric HP0231 is identical to that of monomeric EcDsbA (i.e., CPHC) but different from that of EcDsbC/G (i.e., CGYC/CPYC). Additionally, the *cis*-Pro loop of HP0231 is V*c*P, as in EcDsbA, whereas a conserved threonine residue is found in the *cis*-Pro loops of EcDsbC and EcDsbG. Although the catalytic domain of HP0231 possesses motifs typical for canonical DsbA proteins, evolutionarily it is most closely related to the catalytic domains of DsbG. Similarly, the highly diverged N-terminal dimerization domain is homologous to the dimerization domain of DsbG. The uncommon combination of catalytic and dimerization domains is reflected in the protein's biochemical properties. Some of the properties, such as a redox potential similar to that of EcDsbA, activity in the insulin reduction test (Roszczenko et al., 2012) proved that HP0231 functions in an oxidizing Dsb pathway. On the other hand, HP0231, similarly to EcDsbC or EcDsbG, acts as molecular chaperone, as documented by Lester et al. (2015). HcpE (*Helicobacter* cysteine-rich protein E) containing nine consecutive disulfide bonds, was identified as a substrate of HP0231. HP0231 affects HcpE production and secretion in the native host and also assists its correct folding in *E. coli*. So, due to its dual function HP0231 was named DsbK (Lester et al., 2015).

*H. pylori* does not contain a classical homodimeric DsbC, which in the most Gram-negative bacteria is a periplasmic protein responsible for the rearrangement of incorrectly paired cysteines (Denoncin and Collet, 2013). HP0231 does not complement the deficiency of DsbC in *E. coli* cells as measured by the copper sensitivity assay (Roszczenko et al., 2012). Lack of isomerization activity in *E. coli* cells may result from the inability of HP0231 to interact with EcDsbD. Also, *H. pylori* does not encode a classical DsbD. Instead it has a shortened version of DsbD, CcdA (HP0265) consisting only of the β transmembrane domain of DsbD (Cho et al., 2012). It should be noted at this point that *H. pylori* encodes other protein of the Dsb family—HP0377 with structure similar to CcmG (cytochrome c maturation), previously annotated as DsbC-like protein (Kaakoush et al., 2007). Its biochemical analysis indicates that it might be also involved in Dsb isomerization/reduction pathway (Yoon et al., 2013) (for details see below). It is unclear how HP0231 is re-oxidized *in vivo*. *H. pylori* does not encode a classical DsbB, though it encodes a DsbB-like protein, HpDsbI (HP0595). Mutation of the *hpdsbI* gene results in the appearance of HP0231 in a reduced form, but the active-oxidized form remains more pronounced (Roszczenko et al., 2012), which may suggest that HP0231-DsbI cooperation is rather collateral.

**Figure 5:**
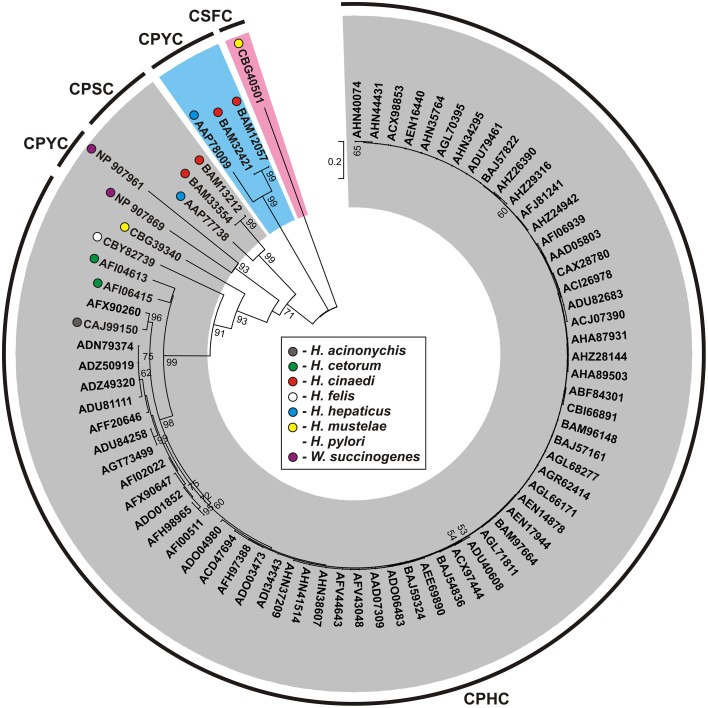
**Phylogenetic tree of the HP0231-like proteins of** ***Helicobacter*** **spp. and** ***Wolinella succinogenes*** **DSM 1740**. The analysis was based on 77 protein sequences and 265 amino acid positions (highly variable portions of the alignments were eliminated by the use of G-blocks). The unrooted tree was constructed using the neighbor-joining algorithm. The statistical support for the internal nodes was determined by 1000 bootstrap replicates. Values of >50% are shown. Accession numbers of the protein sequences used for the phylogenetic analysis are given. The three main clusters were distinguished with gray, pink, and blue color, respectively. The conserved catalytic motifs (CXXC) are presented.

Our bioinformatic *in silico* analysis performed on 71 *Helicobacter* spp. genome revealed a high conservation of the mechanism responsible for protein oxidative folding within two subdivisions of the *Helicobacter* genus, the first subdivision including gastric species and the second including enterohepatic species (with the exception of *H. mustelae*) (Gupta, 2006; Porcelli et al., 2013). All members of the former group contain a dimeric oxidoreductase homolog of the HP0231 of *H. pylori* 26695, with an active site that includes the CPHC motif paired with V*c*P. Potentially, these oxidoreductases interact with DsbIs, although the mechanism of the process is not completely clear. Interestingly, all enterohepatic species and *H. mustelae* lack DsbI, and at the same time, they encode two homologs of HP0231 with various CXXC motifs (CPSC and CPYC in the case of *H. cinaedi* and *H. hepaticus*, and CPHC and CSFC in the case of *H. mustelae*). In every case, the CXXC motif is paired with V*c*P. The observed divergences may reflect differences in the colonized ecological niches that result in various sets of Dsb substrates (Figures 2, 5, Table S1).

The Dsb oxidative pathway of *W. succinogenes* seems to act in a way similar to that of the entrohepatic *Helicobacter* spp. It also encodes two homologs of HP0231 and lacks DsbI. However, the two *W. succinogenes* homologs of HP0231 contain active sites with identical motifs: CPYC paired with V*c*P and were grouped together, within cluster III (Figures 2, 5, Table S1).

## Significant features of bacterial cytochrome c biogenesis

In the highly oxidizing environment of the periplasm, there is a need for selected proteins to be kept in a reduced form, such as c-type cytochromes that are essential for energy metabolism. The cytochrome c maturation process requires the ligation of heme to reduced thiols of the CXXCH motif of the apocytochrome (Thony-Meyer and Kunzler, 1997).

**Figure 6:**
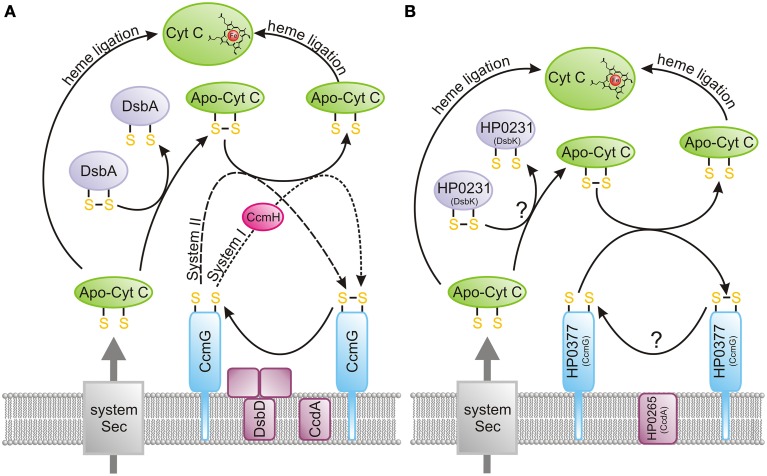
**Cytochrome c biogenesis**. The heme transport mechanism, which is more complex for system I (CcmA-E) compared to system II (CcsA and CcsB or CcsAB), is not shown on the figure. **(A)** Model presenting the bacterial cytochrome c biogenesis (systems I and II). Apocytochrome c, just after its transport across the membrane by Sec proteins, is oxidized by the DsbA/DsbA-like oxidoreductases. Subsequently, it is reduced by CcmG and ligated to heme. Potentially, some apocytochrome c molecules escape oxidation, and they attach heme after they enter the periplasm. CcmG is kept in a reduced form by the CcdA/DsbD-like protein, which transfers electrons from the cytoplasm to the periplasm. **(B)** Model presenting the *Helicobacter pylori* cytochrome c biogenesis (system II). Apocytochrome c, just after its transport across the membrane by Sec proteins, is oxidized by the dimeric oxidoreductase, HP0231 (DsbK). Subsequently, it is reduced by HP0377 (CcmG) and ligated to heme. Potentially, some apocytochrome c molecules escape oxidation, and they attach heme after they enter the periplasm. HP0377 is kept in a reduced form by the DsbD-like protein HP0265 (CcdA), which transfers electrons from the cytoplasm to the periplasm.

Since DsbA or other periplasmic dithiol-oxidases randomly introduce disulfide bonds into apocytochromes, bacteria have evolved a special redox system to revert these disulfides into reduced cysteine residues while in a highly oxidizing environment. Among the five cytochrome c biogenesis systems identified so far, system I (named the Ccm—cytochrome c maturation process) and system II (designated as the Ccs—cytochrome c synthesis) are the most common pathways in the prokaryotic world (Kranz et al., 2009; Sanders et al., 2010; Simon and Hederstedt, 2011). Figure 6A presents the model of bacterial cytochrome c biogenesis. They comprise two kinds of proteins: those involved in the handling of heme that play a role in its ligation to the apocytochrome, and those contributing to reduction of the disulfide bond of the CXXCH heme-binding motif (Kranz et al., 2009).

The main difference between system I and II is the process of heme transport and its ligation into reduced apocytochrome, which appears to be more complicated for system I. In *E. coli*, which uses system I, the CcmABCDEFGH proteins are involved in this process. In contrast, in system II a maximum of four protein components (CcsA, CcsB, CcdA and CcsX) seem to be necessary to achieve cytochrome c biogenesis (Simon and Hederstedt, 2011). The best known system II is the Gram-positive model organism *Bacillus subtilis*. The *B. subtilis* system contains four proteins, ResA (CcsX), ResB (CcsB), ResC (CcsA), and CcdA, of which ResB and ResC are two separate polypeptides that form a complex to deliver the heme and function in the periplasmic cytochrome c-heme ligation (Erlendsson et al., 2003; Colbert et al., 2006; Lewin et al., 2006). In some microorganisms, *ccsA* and *ccsB* are fused into one large ORF (called *ccsBA*), whose product contains 10 transmembrane domains (Frawley and Kranz, 2009) (see below). In both systems, there is a need for the reduction of the disulfide bond of the apocytochrome c heme-binding motif (Bonnard et al., 2010). This is accomplished by the action of CcmG (also known as DsbE for system I, ResA for *B. subtilis* and CcsX for other bacteria). CcmG proteins, both in systems I and II, are kept in the reduced form by an integral membrane protein DsbD, or its shorter analog CcdA. Both proteins catalyze the transfer of electrons from cytoplasmic thioredoxin across the inner membrane to the periplasm. As mentioned above, DsbD consists of eight transmembrane segments (β domain), an N-terminal (α domain) and a C-terminal domain (γ domain). Both, the N- and C-terminal domains face the periplasm (Stirnimann et al., 2006; Rozhkova and Glockshuber, 2008). CcdA is a shorter version of DsbD, consisting only of the β transmembrane domain of DsbD. In contrast to DsbD, which transfers reducing potential to a large numbers of extracytoplasmic proteins, CcdA was thought to only be involved in the cytochrome c maturation process (Katzen et al., 2002). However, some recently published data have shown that in *B. subtilis* or *B. anthracis*, CcdA also plays a role in sporulation and virulence (Erlendsson et al., 2004; Han and Wilson, 2013). Recently a new class of DsbD proteins named ScsB, with a domain organization similar to but not identical with that of DsbD, has been described. However, the role of this class of proteins in CcmG reduction was not analyzed (Cho et al., 2012).

Many studies have tried to decipher the cooperation among periplasmic Dsb proteins during c-cytochrome maturation. Initially, it was thought that introduction of the disulfide bonds into the CXXCH motif of the apocytochrome c, just after its transport to periplasm by the Sec system, was an obligatory step of the cytochrome c maturation process because *dsbA* and *dsbB* mutants in *E. coli* were unable to produce cytochrome c (Metheringham et al., 1996; Sambongi and Ferguson, 1996). However, some recent data in the literature contradict this scheme. A lack of the Dsb proteins in the oxidative pathways in *B. subtilis* or *Rhodobacter capsulatus* suppresses the cytochrome c deficiency of *ccmG* or *ccdA* mutants (Erlendsson and Hederstedt, 2002; Deshmukh et al., 2003; Turkarslan et al., 2008). It was also demonstrated that heterologous expression of the CcsBC of *H. pylori* or CcsAB from *Bordetella pertussis*, which both encode cytochrome c synthetase, in *E. coli* lacking its own cytochrome c machinery results in c-type cytochrome formation. However, the observed effect was significantly enhanced by addition of exogenous reductant (Feissner et al., 2006; Goddard et al., 2010). Detailed analysis of the *Paracoccus denitrificans* cytochrome maturation led to the conclusion that apocytochrome undergoes a competing process of either heme attachment to, or oxidation of, its cysteine thiols (Mavridou et al., 2012).

## Apocytochrome c reduction in *Helicobacter* spp. and *Wolinella* spp.

Our knowledge about the cytochrome c biogenesis processes operating in *Epsilonproteobacteria* is still limited. There are significant differences among particular members of this class of bacteria, mainly with respect to proteins involved in electron transport from the cytoplasm to the periplasm (DsbD vs. CcdA) (Figure 2, Table S1).

The best described system is that operating in *H. pylori* cells. This microorganism uses HP0377 (a lipoprotein anchored in the cytoplasmic membrane of the cell) for apocytochrome c reduction. HP0377 is a thioredoxin-fold protein containing the CSYC motif, which indicates that it functions as a disulfide oxidoreductase. Although there is no direct evidence that HP0377 is involved in cytochrome c assembly *in vivo*, this is likely because its resolved structure is similar, but not identical, to that of other CcmG proteins and because it is able to reduce the oxidized form of apocytochrome c *in vitro* (Yoon et al., 2013). Additionally, *hp0377* is co-transcribed with the *ccsBA* (*hp0378*) gene that is involved in heme transport and its ligation to apocytochrome c (Frawley and Kranz, 2009; Sharma et al., 2010). *H. pylori* or *H. hepaticus* CcsBA, together with *E. coli* DsbD/DsbC, can complement an *E. coli ccm* genes deletion (Goddard et al., 2010). Figure 6B presents the model of *H. pylori* cytochrome c biogenesis.

HP0377 (CcmG) is potentially re-reduced by HP0265 (CcdA). As *H. pylori* possess several proteins containing non-consecutive disulfide bonds and lacks the classical DsbC/DsbG, it was postulated that HP0377 is a multifunctional protein involved in the Dsb isomerization process, which contrasts with most CcmGs that are only involved in the cytochrome c maturation process. HP0377 is the first described CcmG protein having an acidic pKa for the N-terminal cysteine of the CXXC motif, similar to the pKa of EcDsbA or EcDsbC; at the same time, HP0377 presents a low redox potential characteristic of a reductant (Yoon et al., 2013). Undoubtedly, further biochemical, genetic and structural experiments are required to clarify the functioning of the atypical Dsb network of *H. pylori*.

According to our bioinformatics data, an identical cytochrome biogenesis system is potentially active in all of the *Helicobacter* genus members analyzed so far, where a CcmG protein that is homologous to HP0377 is responsible for apocytochrome c reduction. As the CXXC and *cis*-Pro motifs significantly influence the redox properties of the Dsb oxidoreductases, we compared the active sites of CXXC motifs present in various members of *Helicobacter* genus and found that they are strongly conserved (Figure 2, Table S1). All proteins retain Y as the second amino acid of the dipeptide and S as the amino acid most often present in the second position of the motif. Additionally, the *H. pylori* CcmG proteins are paired with T*c*P, as is seen for many other oxidoreductases acting as reductants. However, it should be emphasized that dipeptides of the CXXC present in other CcmGs are extremely divergent (Edeling et al., 2002). In all of the analyzed *Helicobacter* strains, the CcmG proteins are potentially re-reduced by CcdA, and all the strains encode large CcsBA proteins that potentially act as cytochrome c synthetase (Figure 2, Table S1).

There is only one genome of *Wolinella* spp. that has been sequenced so far. Its analysis indicates that *W. succinogenes* also uses the system II cytochrome biogenesis; however, it is more expanded than that of *H. pylori*. It encodes one homolog of HP0377 (CIYC paired with T*c*P motif) and two homologs of EcCcmG, with CPPC/I*c*P and CLSC/I*c*P motifs (Figure 2, Table S1). All of these homologs are potentially re-reduced by the CcdA protein. *Wolinella* also encodes three CcsBA-type CCHL isoenzymes (Nrfl, CcsA1, and CcsA2), which differ in their specificity for apocytochromes, as they recognize various heme c binding motifs (Kern et al., 2010). It is likely that the three CcmGs interact with specific apocytochrome c substrates. As *Wolinella* does not encode DsbC, it is likely that at least one of the CcmGs may be also involved in the disulfide bond isomerization process.

## Apocytochrome c reduction in *Campylobacter* spp. and *Arcobacter* spp.

Most members of the *Campylobacter* genus possess system II cytochrome c biogenesis systems. However, in contrast to the *H. pylori* the cytochrome c biogenesis, those systems resemble the cytochrome c biogenesis system present in *B. pertussis* as the CcmGs are re-reduced by DsbDs (Kranz et al., 2002; Feissner et al., 2005). In the proteomes of *Campylobacter* cells, heme is transported through the cytoplasmic membrane and ligated into cytochrome c by a large protein that resulted from the fusion of CcsA and CcsB, as is seen in all genera of *Epsilonproteobacteria*. In some strains, we found more than one CcsBA, which is also noticeable in the *W. succinogenes* proteome. The most curious observation derived from *in silico* analysis is the presence of putative CcmGs of different origins. In some strains, there is more than one gene encoding this protein per genome. The majority of *Campylobacter* CcmGs are annotated as TlpA. We noticed that they are related to TlpA (*t*hioredoxin-*l*ike *p*rotein) and to CcmG, and they potentially belong to the TlpA/ResE/CcmG subfamily (Marchler-Bauer et al., 2015). Some members of *C. coli* species possess two CcmGs: one is related to the *H. pylori* CcmG (HP0377 in the *H. pylori* 26695 genome), and the second one is a member of the TlpA/ResE/CcmG subfamily. TlpA was identified for the first time in *Bradyrhizobium japonicum*, a nitrogen-fixing soil bacterium, as a Dsb protein involved in the biogenesis of apocytochrome aa_3_ (Loferer et al., 1993). Similar to the classical CcmG, it is anchored to the cytoplasmic membrane and its C-terminal domain contains a thioredoxin domain exposed to the periplasm. Its structure revealed several atypical features that are reflected in the biochemical properties of the protein (Capitani et al., 2001). Analysis of TlpA function in *B. japonicum* and *Neisseria gonorrhoeae* revealed that TlpA is a multifunctional oxidoreductase (Achard et al., 2009; Mohorko et al., 2012). In CcmG proteins the critical residues of CXXC motif fluctuate considerably. However, it should be noted that in most cases *Campylobacter* genus representatives retain the proline as a second amino acid of the XX dipeptide in CXXC motif and I*c*P motif. When more than one CcmG protein is present in most cases CPSC motif is paired with T*c*P and CGPC/CSPC motif is paired with I*c*P. This preliminary analysis suggest differences in the recognized substrates. Evidently, the function of CcmGs in *Campylobacter* spp. requires experimental investigation, as all bioinformatics predictions should be verified. The cytochrome biogenesis system II of *Arcobacter* spp. seems to act similarly to bacteria in the *Campylobacter* genus. However, all the CcmGs of *Arcobacter* spp. show homology to EcCcmG (Figure 2, Table S1).

## Role of *Epsilonproteobacteria* Dsb proteins in pathogenesis, and potential therapeutic and prophylactic applications

The virulence of many bacterial pathogens depends on extracytoplasmic proteins, many of which contain two or more cysteine residues and achieve their final structure as a result of disulfide bond formation catalyzed by the Dsb proteins. Thus, inactivation of the Dsb system very often results in attenuation of bacterial pathogenicity (Raczko et al., 2005; Heras et al., 2009). Also, although members of *Epsilonproteobacteria* analyzed so far that are defective in the Dsb oxidative or isomerization pathways do not exhibit growth inhibition, they very often display attenuated virulence in animal models. The *hp0595* mutated *H. pylori* revealed a greatly reduced ability to colonize mice gastric mucosa (Godlewska et al., 2006). Also, it was shown that CjDsbA1 (the main periplasmic oxidoreductase of *C. jejuni*) plays a crucial role in motility and autoagglutination, mechanisms that are necessary for the bacteria to colonize and spread within a host organism (Grabowska et al., 2014). Moreover, the inactivation of two membrane *Campylobacter* oxidoreductases (DsbB and DsbI) resulted in significant attenuation of chicken intestine colonization by *C. coli* 23, and also reduced the invasion/intracellular survival abilities (in the T84 cell line) of the *C. jejuni* 81-176 strain (Lasica et al., 2010).

The emergence of “new” bacterial pathogens and fast-spreading multidrug resistant strains underscores the urgent need to develop novel therapeutic agents, as well as novel diagnostic and prophylactic tools to fight infectious diseases. Broad knowledge about virulence mechanisms of bacterial pathogens and their interactions with the host, combined with advances in structural biology in the past decade, should help achieve this goal. It is generally accepted that virulence factors are significant targets for therapeutic drugs. Proteins of the Dsb system, which play a key role in the virulence of many pathogenic Gram-negative organisms, represent possible new drug targets (Heras et al., 2015). Their localization in the bacterial periplasm renders them easily accessible to potential small-molecule inhibitors. Furthermore, use of Dsb-protein inhibitors should not create selective pressure for developing resistance. Recently research on the Dsb system of *E. coli* has resulted in extensive knowledge about protein structure and mechanism of action, and that has enabled the design of specific inhibitors of Dsb protein activity (Duprez et al., 2015; Halili et al., 2015).

## Conclusions

Comparison of the Dsb systems of *C. jejuni* and *H. pylori*–two well characterized members of *Epsilonproteobacteria* revealed enormous diversity of the mechanisms involved in oxidative protein folding. The functioning of the *Arcobacter* and *Wolinella* genera Dsb systems has so far been poorly understood. Given the importance of disulfide bond formation to achieve native protein structures and the diversity of disulfide bond formation oxidative pathways in bacteria, further research in this field will add to our biochemical and microbiological understanding of these pathways. Additionally, as proteins of the Dsb system play a key role in virulence, they represent possible new drug targets. Inhibition of the Dsb protein interaction with it protein substrates or redox partners could block the formation of virulence factors. An understanding of *Epsilonproteobacteria* Dsb protein structures and their activities may facilitate discovery of an effective drug against these important human pathogens.

### Conflict of interest statement

The authors declare that the research was conducted in the absence of any commercial or financial relationships that could be construed as a potential conflict of interest.

## Supplementary material

Table S1 **Distribution of Dsb-like and associated proteins (with their accession numbers) in *Arcobacter* spp., *Campylobacter* spp., *Helicobacter* spp., and the *Wolinella succinogenes* DSM 1740 proteomes**.
